# Tackling the blind spot of poor-quality medicines in Universal Health Coverage

**DOI:** 10.1186/s40545-020-00208-4

**Published:** 2020-05-11

**Authors:** E. S. F. Orubu, C. Ching, M. H. Zaman, V. J. Wirtz

**Affiliations:** 1 Institute for Health System Innovation & Policy, Boston University, Boston, MA USA; 2 Department of Biomedical Engineering, Boston University, Boston, MA USA; 3 Department of Global Health, Boston University School of Public Health, Boston, MA USA

**Keywords:** Poor-quality, Substandard, Falsified, Medicine, Quality assurance, Universal Health Coverage

## Abstract

**Background:**

Universal Health Coverage (UHC) is challenged by the prevalence of poor-quality medicines, those that either do not meet required specifications (substandard) or are outrightly fraudulent (falsified), especially in Low- and Middle-Income Countries, LMICs. Whereas poor-quality medicines are a significant burden in these countries, medicine quality still remains a neglected component of UHC programs. This article describes key barriers to quality medicines and presents five select approaches leveraging the scale-up of UHC for medicine quality assurance.

**Main body:**

Barriers to medicine quality assurance, while numerous, are described in five key inter-related domains as: low political priority, weak regulatory systems capacity, poor access to accredited facilities and licensed outlets, medicine manufacturing and other supply-chain challenges, and lack of public awareness. Five select approaches for leveraging the scale-up of UHC for medicine quality assurance in LMICs are (1): political commitment (2) strengthening the capacity of regulatory authorities and investment in detection technologies as part of national security (3); licensing of medicines outlets and expanding pharmacovigilance (4); strengthening the supply-chain; and (5) public awareness and participation.

**Conclusions:**

Unchecked, poor-quality medicines can jeopardize UHC. National governments in LMICs need to prioritize medicine quality assurance through enforcing policies, regulatory strengthening and investments in technologies. Healthcare facilities and insurance schemes under UHC also play critical roles through incorporating medicine quality assurance into procurement practices and by promoting awareness among beneficiaries. Tackling medicine quality with a committed systems approach will enhance progress towards UHC implementation.

## Background

In 2015, Universal Health Coverage (UHC) was declared a development target under the 17 United Nations’ Sustainable Development Goals (SDGs). SDG 3 on health includes target 3.8 which seeks to “Achieve universal health coverage, including financial risk protection, access to quality essential healthcare services and access to safe, effective, quality and affordable essential medicines and vaccines for all”. UHC is, thus, a shared vision to ensure equity, quality and financial security in access to healthcare by 2030 [1].

While access to quality medicines is mentioned under SDG target 3.8, medicine quality itself is not one of the indicators of progress towards UHC. Currently, the only indicator for medicines is “Proportion of health facilities that have a core set of relevant essential medicines available and affordable on a sustainable basis” [2, 3]. Access to medicines, however, is a 5-dimensional concept that includes availability and affordability, with quality at its core [4–6].

A poor-quality medicine, as defined by the World Health Organization (WHO), is one that is either substandard or falsified (SF). A substandard medicine fails to meet the required specifications – usually, but not limited, as to content of active ingredient(s), impurity limits, microbiological activity, and dissolution profile. Falsified medicines are “medical products that deliberately/fraudulently misrepresent their identity, composition or source” [7]. Poor-quality medicines have serious consequences that can slow progression towards UHC. One especially pressing consequence is Antimicrobial Resistance (AMR) development, which itself can undermine the SDG goals [8–10]. Thus, the ability to ensure medicine quality throughout the supply chain is integral to the implementation and success of UHC, especially for Low- and Middle-Income Countries (LMICs).

In 2018, the WHO estimated a 10.5% prevelence of SF medicines in the supply chain in LMICs; though the problem is worldwide given global manufacturing and supply chains, the rise in internet purchasing of medicines, and forced migration [11–14]. With an estimated USD 30.5 billion cost, poor-quality medicines are a significant economic burden in these countries [15].

However, despite its importance, medicine quality as an essential element of UHC is largely ignored and a “blind spot” of health sytems strengthening. Acknowledging this importance and understanding the barriers to medicine quality assurance can help countries more efficiently scale-up UHC. Recently, the United States Pharmacopoeia commissioned a report on poor-quality medicines focusing on the implications for health systems [16]. Our review complements the report by focusing on strategies to address medicine quality assurance challenges in LMICs.

The aim of this paper is to present a set of considerations at national and international levels for medicine quality assurance within UHC in LMICs. After briefly summarizing the burden of and barriers to medicine quality, the paper focuses on approaches with in the scale-up of UHC to address poor-quality medicines.

### Poor-quality medicines: counting the cost

A review of modelling studies show that poor-quality medicines can result in serious harm and large inefficiencies which can be pitfalls to UHC, as shown in Tables 1 and 3 [15, 17–22]. The estimated economic losess from SF anti-malarials alone constitute a significant portion of the national budget for impacted countries (Table 1). In Congo, for example, the estimated USD 151 million loss equals 2.5% of the USD 5.9 billion 2019 national budget [21, 23] . In Uganda, the USD 31 million loss is about 5% of the USD 637 million Health Budget for 2019/2020 [22, 30]. These impacts on the budget, as well as health and cost implications as comprehensively illustrated by Ozawa et al., 2019, demonstrate that poor-quality medicines are a drain on health systems in LMICs [16]. These inefficiences jeopardize progress towards UHC.

**Table 1:** Impact of poor-quality medicines from modelling studies (as obtained from a rapid-review of published literature)

No	Region/Country	Age-group	Disease condition	Therapeutic group of medicine	Estimated prevalence of poor-quality medicines, %	Impact		Source (Ref.)
						Estimated deaths, median	Economic costs, USD million, 2017	
**1**	Sub-Saharan Africa	Under-5	Malaria	Antimalarial	0–40	122,350^a^	ND^b^	[17]
**2**	Global	Under-5	Acute LTRI^c^/Pneumonia	Antimicrobials	10	72,430–169,271^d^	ND^b^	[15]
	Sub-Saharan Africa	Under-5	Malaria	Antimalarial	7.6^e^	116,000^f^	38.5^f^	
**3**	LMICs^g^	ND	ND	All	13.6	ND	31250^h^	[18]
**4**	LMICs	All	Tuberculosis	Anti-TB^i^	6.7^j^	255,115	ND	[19]
	LMICs	All	HIV/AIDS	Anti-Retroviral	4.2^j^	72,183^k^	ND	
**5**	Nigeria	All	Malaria	Antimalarial	12–50^l^	12,300^m^	892^m^	[20]
**6**	Congo (DRC)^n^	Under-5	Malaria	Antimalarial	19	10,370^m^	151^m^	[21]
**7**	Uganda	Under-5	Malaria	Antimalarial	21–31^o^	1100^p^	31	[22]

^a^Median for 2010

^b^*ND* not determined/not specified

^c^*LRTI* lower respiratory tract infections

^d^The figures refer to excess deaths for 2010. The lower figure is the estimate for when the antimicrobials have reduced activity while the higher figure is the case for no activity

^e^Artemisinin Combination Therapies, ACTs, only

^f^Median value for the higher of two alternative estimates

^g^Low- and middle-income countries

^h^Median market size of substandard and falsified medicines. Data as reported from eight studies dating from 2003 to 2017

^i^Anti-Tuberculosis

^j^Median prevalence rate for the therapeutic group of medicine

^k^Lost disability-adjusted life years (DALYs) rather than mortality

^l^Variable depending on antimalarial class: 12% for ACTs, 50% for chloroquine and other treatments

^m^Annual mean values

^n^Democratic Republic of Congo

^o^Variable depending on antimalarial class: 21% for ACTs, 22% for quinine and 31% for other treatments

^p^Additional annual deaths due to SF antimalarial medicines

### Barriers to medicine quality assurance in LMICs and implications for UHC

There are numerous barriers to medicine quality assurance in LMICs [31–37]. Using a construct based on a systems thinking approach, these can be grouped into five key inter-related domains as:

I. low political priority,
II. weak regulatory systems capacity,
III. poor access to accredited facilities and licensed outlets,
IV. medicine manufacturing and supply-chain challenges, and
V. lack of public awareness.
I. *Low political priority*

Low political priority for SF medicines within UHC is a key barrier to medicine quality assurance. Low political priority encompasses several factors at the national and international levels. In international discussions about UHC, medicine quality has not received priority for many reasons. These include, but are not limited to, the focus on financial considerations with regards to access to medicines and UHC coverage. Another reason is that the negative health impact of poor-quality medicines is often insidious and complicated due to underlying health conditions of those affected that may result in poor outcomes that manifest over longer time frames. In addition, there is a lack of surveillance data on mortality and morbidity due to poor-quality medicines in many LMICs. As a result, politicians deprioritize quality over other apparently more tangible programs [35, 38]. Corruption and other governance failures have also been mentioned [36]. Collectively, these factors account for a lack of urgency or commitment at the political/policy level.

II. *Weak regulatory systems capacity*

Poor-quality medicines tend to be prevalent in settings where constrained access to medicines coexists with poor governance and weak regulatory capacity [7]. Constrained access implies high demand in settings with high disease burdens, limited ability to pay, and the ability to purchase almost all medicines over the counter. The weak regulatory structures in most LMICs mean that many countries do not have the capacity to prevent poor-quality medicines entering the supply chain. As of 2018, fewer than 30% of countries were assessed as having a Stringent Regulatory Agency, SRA, which is a “regulatory system operating at advanced level of performance and continuous improvement” (Level 4) [39, 40]. The absence of Level 4 SRAs, or WHO-Listed Authorities at Maturity Levels 3 or 4, the new nomenclature, is, thus, another key barrier to medicine quality assurance in LMICs.

III. *Poor access to accredited facilities and licensed outlets*

In many LMICs, the first port-of-call for people seeking medical treatment is often a medicine outlet – pharmacy or drug-store. Access issues – affordability, time, medicines availability, high patient copayments – are often the main reason people visit medicine outlets, licensed and unlicensed, instead of hospitals. While pharmacies are usually licensed, most drug-stores are not [41, 42]. Additionally, in rural areas, these medicine outlets may be the only “healthcare facility” readily available. However, unlicensed medicines outlets are associated with a high risk of poor-quality medicines [43].

IV. *Medicines manufacturing and other supply-chain challenges*

Not many LMICs have the manufacturing capacity to produce high-quality medicines. For instance, Nigeria manufactures only about 30% of pharmaceuticals (drugs) needed, mostly due to high production costs [44]. In an attempt to reduce costs, manufacturers may be forced to procure active pharmaceutical ingredients (API), usually the most expensive part of a medicine, from suppliers based on offered price rather than on quality [45]. A low manufacturing capacity for finished pharmaceutical products also means that most medicines would need to be imported, which can lead to the import of poor-quality medcines. In LMICs that do have a high local manufacturing capacity, quality may not always be assured, or the focus of the NRA may not be on quality assurance because of capacity challenges [46, 47]. For example, in Pakistan, a study of twenty-seven ibuprofen API samples used by manufacturers in the country found that 81.5% (22/27) failed to comply with pharmacoepial specifications [48]. Tellingly, the WHO database of pre-qualified pharmaceutical manufacturers, assessed as meeting Good Manufacturing Practice, for defined essential medicines lists (for LMICs) has only one manufacturer each from Bangladesh, Egypt, Kenya and Morrocco, with the bulk from China and India [49].

The downstream supply-chain for many LMICs is complicated with many formal and informal players, or licensed and unlicensed outlets. For example, there are about 100,000 licensed and 100,000 unlicensed medicine outlets in Bangladesh [50]. In Nigeria, a census in 16 (out of 36) states showed that only 13% of non-pharmacist manned medicine outlets, or patent medicine vendor outlets, were registered with the statutory regulatory authority [51]. These complexities makes effective regulation even more difficult [52–55].

In some settings where public procurement regulations stipulate that the tenders are awarded on prices only, pressure is created on administrators of healthcare facilities to purchase medicines based on price without requirements for quality assurance [35, 45]. Generally, inefficiencies in the supply-chain contributes to a proliferation of poor-quality medicines [31].

V. *Lack of public awareness*

A lack of, or low, public awareness of the dangers posed by poor-quality medicines is a critical barrier to medicine quality assurance. The patient/client who procures a drug from any medicine outlet does so in the belief that the medicine is of quality, safe and effective. In this belief, the patient relies on the staff at this outlet to be knowledgeable enough and to have the “right” medicine for the complaint. Often, where the medicine outlet is unregistered, or unmanned by a professional, this is not the case [43]. Irrational dispensing at these non-professionally manned medicines outlets can compound the problem [56].

### Approaches to medicine quality assurance

To overcome these barriers, interventions that include a whole-of-system approach to medicine quality assurance targeting all stakeholders from policy-makers to medicine outlets are required. Here, we present five select approaches leveraging the scale-up of UHC for medicine quality assurance.

I. *Political commitment: enacting and enforcing regulations*

Political committment may be the most potent lever to tackling poor-quality medicines. Countries need to target zero tolerance for SF medicines as they implement or expand UHC. For this, political commitment is required on several fronts [57]. Penalties for manufacturing and distributing poor-quality medicines need to be prescribed and enforced. For example, in November 2016, Nigeria passed the Counterfeit and Fake Drugs and Unwholesome Processed Foods (Miscellaneous provision) Amendment Bill 2015 with stiffer penalties including life imprisonment and expensive fines [58]. Similarly, in Thailand, the drug act was updated and strenghtened to include stronger fines and prison sentences [59]. In Europe, falsifying drugs also holds strict punishments [60]. For countries that do not have laws in place, the Model Law on Medicine Crime has been developed and proposed for countries to freely adopt to comprehensively strengthen their laws [61]. Once formed, stringent and sustained enforcement of these regulations would be required. One way to do this is the use of combined law enforcement and judiciary task forces that can both arrest and sentence offenders speedily. Bangladesh, for example, have used special military and police task forces, backed by mobile courts, to enforce regulations on poor-quality and expired medicines leading to closures of manufacturers, wholesalers/distributors and health facilities; as in the Phillipines [62, 63]. In Nigeria, a renewed political will, higher fines, and enforcements lead to an estimated 80% drop in SF medicines from 2001 to 2006 [64–66]. Evidence for stringent enforcement of legislations as an effective intervention to ensuring medicine quality can also be seen from related health system interventions designed to reduce irrational antibiotic prescription and use in LMICs [67, 68].

Political commitment extends beyond manufacturing and distributing poor-quality medicines, to supporting and enforcing all other approaches presented. This includes policies mandating reporting on the results of implementation for the described approaches to tackling poor-quality medicines [69, 70]. These will be critical in progression towards UHC, by providing baseline data against which progress and inteventions can be measured. Measuring access to medicines assured for quality in a health system as part of reporting of progress towards UHC acknowledges the critical contribution of this dimension and allows for appropriate resource allocation [71]. Such policies demonstrate prioritization by governments of the SF challenge. Results can also allow governments to obtain credit for successful policy implementation and increase trust in the health system.

II. *Regulatory system strengthening and investment in technologies as part of national security*

Capacity building at the local NRAs is imperative as part of UHC. Some considerations for regulatory strengthening are provided by Roth et al. [72]. One effective way for governments to mobilize resources to strengthen the capacity of regulatory authority would be to consider such investment a national security agenda as poor-quality medicines affects all aspects of national development.

Investments in technologies for the detection of SF medicines throughout the medicines supply chain can be a support in countries with weak regulatory systems to identify poor-quality products. There are many technologies for the screening of poor-quality medicines with varying costs and utility [73]. While the costs for aquiring these differ according to their capability and portability, or depolyability along nodes of the medicines supply chain, as illustrated in Table 2, a cost-effectiveness analysis for Lao People Democratic Republic found that investments for any of six selected portable or field-use technologies with unit purchase costs of $3 - 1,400 was cost-effective for the detection of falsified products by the NRA [74].

**Table 2:** Estimated costs of selected commercially-available technologies for the determination of medicine quality

	Device	Applications/Feature	Unit Price (USD), estimated, 2017
**1**	TruScan®	Pharmaceutical raw materials. Hand-held (portable)	70,000^a^
**2**	HPLC	Identity, impurities, content uniformity	80,000^b^
**3**	UV-Vis spectrophotometer	Identity, quantification. Bench-top	10,000^c^
**4**	Mini-Lab®	Identity, semi-quantification. Portable	8000^a^
**5**	Dissolution apparatus	Dissolution; bench-top	10,000^d^

^a^Manufacturer’s quoted price. 2017. Private communication. (Price includes installation and training)

^b^Estimated based on an internet search on Amazon

^c^Purchase price for a double-beam brand. 2018

^d^Indicative price for an entry-level, used, system

Investments in technology remain cost-effective in a task-shifting scenario (Table 3). In Congo (DRC), equipping its 400 General Hospitals (secondary health facilities) with a UV-Vis Spectrophotomer and a dissolution apparatus each would cost an estimated USD 8 million, or 5% of the simulated USD 151 million loss due to poor-quality antimalarials in two provinces [21]. Even allowing for an estimated annual consumables and analyst (pharmacist) salary, this investment remains cost-saving. For all three countries of Congo, Uganda and Nigeria, for which modelled estimates of the impact of SF medicines are available, investments in technology to check SF medicines would cost at most 1% of the 2019 budget. Thus, compared to the economic losses, investments made under any of these scenarios represent “savings” of at least 1% of the budget from losses that would otherwise have resulted from poor-quality antimalarials. Countries without the resources to deploy technology may choose to request for assistance from international programs. International agencies should also consider voluntary investments in technologies for detection of SF medicines, as well as capacity building, in LMICs [75].

III. *Improving access: licensing of medicines outlets under UHC.*

**Table 3:** Investments in selected technologies for the detection of SF medicines under three investment scenarios for all public secondary or tertiary hospitals cost at most 1% of the 2019 budget and, at a maximum of 0.3% of estimated economic losses due to SF antimalarials, represent savings for Congo, Nigeria and Uganda

Country	Equipment cost scenarios		Health facilities, n	Fixed cost [equipment cost for all health facilities], USD million	Pharmacist salary^,e,f^ USD million	Consumables USD million	Repeating Annual cost [salary+ consumables] USD million	Total cost (fixed cost + annual cost) USD million	Total cost (% of 2019 budget)	Total cost (% of estimated economic loss)
	Level^a^	Cost USD								
**Congo**										
	High	90,000	400^b^	36	3.4	0.1	3.5	39.40	0.67	0.26
	Medium	20,000	400	8	3.4	0.1	3.5	11.40	0.19	0.08
	Low	18,000	400	7.2	3.4	0.1	3.5	10.60	0.18	0.07
**Nigeria**										
	High	90,000	107^c^	9.6	1.3	0.1	1.4	10.90	0.04^g^	0.01
	Medium	20,000	107	2.1	1.3	0.1	1.4	3.40	0.01	0
	Low	18,000	107	1.926	1.3	0.1	1.4	3.20	0.01	0
**Uganda**										
	High	90,000	65^d^	5.85	0.6	0.1	0.7	6.40	1.01	0.21
	Medium	20,000	65	1.3	0.6	0.1	0.7	1.90	0.30	0.06
	Low	18,000	65	1.17	0.6	0.1	0.7	1.80	0.28	0.06

^a^Costs are derived from Table 2. The levels refer to a ranking from the most expensive to the least among the HPLC (high); UV-Visible spectrophotometer (medium) and low (Mini-Lab). For each of these devices the estimated cost of a dissolution apparatus (USD 10000) was added to give the cost in Table 3

^b^Secondary health facilities [24]

^c^Tertiary health facilities, national [25]

^d^All public facilities – a mix of all 3 levels, primary secondary and tertiary [26]

^e^Starting salaries for pharmacist, annualized [27–29]

^f^Rounded off to the nearest hundred thousand

^g^Nigeria budget for 2019: USD 28.89 billion (Naira 8.83 trillion). Sources: https://www.reuters.com/article/nigeria-budget/update-2-nigerian-president-offers-record-34-billion-budget-for-2020-idUSL5N26T4EA

With increased access to health services in accredited facilities and licensed medicine outlets through scaling up of UHC, there should be less incentive for the public to visit unlicensed/unregulated medicines outlets, which often have poorly-trained staff and distribute poor-quality medicines [76–81]. To protect patients, all such outlets should be proscribed, and so should be open medicine markets with no regulatory oversight. While increasing the number of trained personnel – pharmacists – should be encouraged to meet internationally-defined ratios, competencies in detecting SF medicines, and geographical coverage of rural areas, in the short-term, some training could be provided to non-professionals already manning unlicensed premises to increase access to health services. This approach has been utilized in Tanzania under the Accredited Drug Dispensing Outlets, ADDO, initiative [82]. Under the ADDO in Tanzania, measures to improve medicine quality includes the requirement that only drugs licensed by the local regulatory authority, thus of the right quality, are allowed to be sold. The ADDO model has been replicated in other countries, under different names, such as in Bangladesh, Liberia, and Uganda [82, 83]. However, it should be noted that there are issues with this approach, for example, poor dispensing of medicines for malaria and antibiotics in Tanzania and Kenya with implications for treatment efficacy [84–86]. Thus, these outlets also require an active and strict monitoring strategy [56]. Ultimately, medicine outlets should be manned only by those with professional certification. Mechanisms for ensuring medicine quality in outlets with non-professionals, as well as those manned by professions, would be required. A commitment to improving access under UHC addresses the access barrier and removes the economic incentive for the use of unregulated medicine outlets.

Pharmacovigilance – the active surveillance for adverse effects to medicines including suspected inefficacy resulting from possible poor-quality medicines – could be expanded to all formal healthcare facilities as part of their accreditation to deliver their services under UHC. Though medicine quality issues are not commonly reported in pharmacovigilance reports or alerts, adverse effects reporting could be the first sign of a poor-quality medicine as was the case with the “*My Pikin*” tragedy in Nigeria in which an adulterated cough syrup lead to deaths in children [87]. A formal national roll-out of pharmacovigilance centers in Rwanda with a focus on medicine quality reporting by all stakeholders – patients and healthcare workers – is identified as one of several strategies that led to the low prevalence of poor-quality medicines in 2013 [88].

IV. *Supply-chain strengthening: Quality-focused good procurement practices*

Quality-focused, good procurement practices are one option to overcoming supply-chain challenges. With the scale-up of UHC, it is expected that individual purchase of medicines is replaced by large-scale procurement by public institutions [77]. The increase in procurement of medicines is an important opportunity to incorporate mandatory quality checks by the procurer [89]. In Europe, the Falsified Medicines Directive (FMD), an EU-wide directive to prevent the entry of poor-quality medicines into the medicine supply chain, is an example of an innovative regulation aimed at medicine quality assurance within the supply chain that was introduced in 2019. This policy contains requirements and penalties securing the medicine supply chain against SF medicines. For example, at the manufacturing level, the FMD mandates pharmaceutical manufacturers to encode features that would allow for medicine quality authentication at the point of supply to the patient, as well as make a financial contribution towards this quality assurance mechanism [90, 91]. At the hospital level, a risk-based quality verification system can be used where medicines are “triaged” and tested for quality based on their source before onward distribution in the supply chain. In this system, medicines from known and reputable manufacturers are not tested, but medicines from unknown manufacturers would be tested [91]. The FMD is part of a mandated medicines verification system in countries with established UHC programs in regulated settings that provides security and tracking services to protect the medicines supply chain, from manufacturers to end-users, against poor-quality medicines.

In countries with relatively-weak regulatory systems, procurement agencies can play a critical role in quality assurance. These agencies, and supply chain management systems in general, can include a contracted medicine quality assurance service using either a risk-based or routine random sampling protocol to ensure the quality of procured medicines. For example, public procurement agencies can create partnerships with academic research institutions to establish medicine quality assessments hubs where potential new pharmaceutical suppliers go to obtain medicine quality certification before they could supply medicines. A preliminary screening to limit generics to a maximum of about three for each individual medicine, identified by its International Non-proprietary Name (INN), included in the country’s essential medicines list can help reduce the number of samples that need to be tested. This screening can be conducted using the protocol suggested in the WHO Medicine Quality Assurance System for Procurement Agencies [92, 93]. The contracted service provider should be requested to perform potency and dissolution tests to ensure both active ingredient content and a dissolution profile necessary to ensure bioavailability. Repeat testing following storage may also be necessary. This approach prioritizes quality over cost. In Kenya, the Mission for Essential Drugs and Supplies (MEDS) program run by faith-based hospitals with a WHO-accredited laboratory for the routine and continuous analysis of the quality of procured medicines utilizes a quality-focused procurement model that could be adopted and scaled up under UHC [94, 95]. This model is reported to have resulted in a low percentage (< 5%) of out-of-specification medicines between 2004 and 2008 [96].

Where public and private hospitals employ direct procurement, there is a need for increased vigilance. Within formal health systems, pharmacists take the lead in this, and in order to be fully equipped to respond to suspected issues of poor-quality medicines, should be able to evaluate such medicines using low-cost technologies. Physical/visual inspection checks can also be employed – as part of a three-level approach [92]. Mandating a Quality Control/Quality Assurance officer pharmacist who employs this checklist as a first step to detecting possible poor-quality medicines may be necessary. Payment linked to performance of these officers employed as part of the accreditation of the health facilities providing service under UHC could provide important incentives. These strategies could be complemented by track and trace technologies which has been proven to be effective in combating SF medicines in countries like Turkey, for example [97].

V. *Increased public awareness and participation*

Consumer awareness programs and participation can help to check poor-quality medicines. When beneficiaries join health insurance schemes, they should receive information in their beneficiary package on medicines. This information should include how they can protect themselves and others from purchase of SF products. One such public awareness program initiative was launched in the early 2000s in Nigeria. “*Operation Shine your Eyes*” was a public awareness program ran on mass media by the National Agency for Food and Drug Administration (NAFDAC) aimed at increasing awareness. This campaign sensitized customers to the dangers of SF medicines and led them to request for the Agency’s Registration Number for all medicines bought as a sign of quality. This program was so successful that it was uncommon to buy medicines from a medicine outlet at the time without asking for NAFDAC registration number – as the intervention asked them to [98]. Consumer participation in quality checks is based on the use of mobile authentication services on platforms of third-party providers in which the consumer scratches a panel with a code that they can call or message to confirm product authenticity [58, 99]. Third-party providers should be obligated, under the mandatory reporting policy, to share data with the NRA to increase transparency and public awareness. End-user awareness and participation both within and outside health facilities, thus, is also required.

## Conclusions

In September 2019, at the UN summit on UHC, the WHO made a call for countries to increase spending on primary healthcare by at least 1% of their GDP [100]. A similar commitment, but with a different target percentage, may need to be made for medicine quality assurance as countries implement UHC. There is a need to comprehensively address poor-quality medicines in LMICs striving towards UHC. Poor-quality medicines cause human and economic losses. Understanding barriers to medicine quality assurance can help countries designing or implementing UHC programs avoid the blind spot of poor-quality medicines. Governments need to prioritize medicine quality assurance through policies that enact and enforce regulations, measure and report on quality and strengthen regulatory capacity, as a matter of national security. Insurance schemes under UHC can play a critical part by incorporating quality assurance into organizational procurement practices, accreditation of outlets and education of their beneficiaries. Tackling medicine quality with a committed systems approach will enhance progress towards UHC implementation.

## Data Availability

Not applicable.

## Abbreviations

ACT: Artemisinin-based combination therapy
ADDO: Accredited drug dispensing outlet
AMR: Anti-microbial resistance
API: Active pharmaceutical ingredient
DALY: Disability-adjusted life year
DRC: Democratic Republic of the Congo
FMD: Falsified medicines directive
GDP: Gross Domestic Product
INN: International Nonproprietary Name
LMICs: Low and Middle-Income Countries
MEDS: Mission for Essential Drugs and Supplies
NAFDAC: National Agency for Food and Drug Administration
NRA: National Regulatory Authority
SDGs: Sustainable Development Goals
SF: Substandard and falsified
UHC: Universal Health Coverage
UN: United Nations
USD: United States Dollar
WHO: World Health Organization
